# Use of flavored electronic cigarette refill liquids among adults and youth in the US—Results from Wave 2 of the Population Assessment of Tobacco and Health Study (2014–2015)

**DOI:** 10.1371/journal.pone.0202744

**Published:** 2018-08-23

**Authors:** Liane M. Schneller, Maansi Bansal-Travers, Maciej L. Goniewicz, Scott McIntosh, Deborah Ossip, Richard J. O’Connor

**Affiliations:** 1 Department of Health Behavior, Roswell Park Comprehensive Cancer Center, Buffalo, NY, United States of America; 2 Department of Public Health Sciences, University of Rochester Medical Center, Rochester, NY, United States of America; Duke University, UNITED STATES

## Abstract

**Introduction:**

Flavored e-cigarettes are enticing to new users and established cigarette smokers using e-cigarettes to quit smoking due to the wide variety of flavor options. However, specific flavor combinations that are popular among e-cigarette users are understudied. Recently, the Deeming rule extended the US Food and Drug Administration’s authority over all tobacco products, including e-cigarettes.

**Methods:**

The Population Assessment of Tobacco and Health Study Wave 2 data were analyzed to assess the prevalence of self-reported flavor categories that are used individually and in combination with other flavor categories among past 30-day youth and adult e-cigarette users in the US.

**Results:**

Most youth and adult participants reported using a flavored e-cigarette. Reporting an individual flavor category was more common than reporting a combination of flavor categories. Fruit flavor was the most common flavor category reported among youth, and ‘menthol/mint’ was most common among adults. Fruit and candy/other sweets were the most common flavor categories reported together among both youth and adult past 30-day e-cigarette users.

**Conclusions:**

The use of flavored e-cigarettes is very popular among youth and adults. Most consumers reported using a single flavor category, although some consumers did use a combination of flavor categories. Preference for menthol/mint among adults may represent a carryover from associations with tobacco cigarettes. Typically, sweeter flavors, such as fruit, were among the most popular flavor categories reported, both individually and in combination with other flavors, while more bitter flavors (i.e.: clove/spice) were less common.

**Implications:**

This study identified predominant flavor categories among past 30-day youth and adult e-cigarette users. Findings indicate that the wide variety of flavors available and the freedom to “mix-and-match” flavors may maintain use of e-cigarettes among youth and adults, and future research should focus on the composition of the ENDS liquid/vapor to disentangle the contributions of sweeteners and flavorants.

## Introduction

The passing of the Family Smoking Prevention and Tobacco Control Act of 2009 by the United States (US) Food and Drug Administration (FDA) restricted the use of ‘characterizing flavors’ in cigarettes, but not other tobacco products [1]. Flavored tobacco use is associated with younger age and more favorable perception of tobacco products [2]. Concerns have also been raised about potential health risks from inhaling flavor additives (e.g., cherry and strawberry), which can contain inhalation toxicants and respiratory irritants such as benzaldehyde and benzyl alcohol [3].

In August 2016, the Deeming rule extended FDA’s regulatory authority to all tobacco products including electronic nicotine delivery systems (ENDS), which include e-cigarettes [4, 5]; to date, there has been a lack of action assessing characterizing flavors in these products [6]. The availability of flavored e-cigarettes is enticing to new users as well as established cigarette smokers who are looking for an alternative to traditional cigarettes [3, 7–11]. Many users, primarily youth (ages 12–17) and young adults (ages 18–24), report the availability of flavors as a reason they use e-cigarettes [7]. Adult e-cigarette users report using flavored e-cigarettes because it increased their satisfaction, created a better feel and taste, and allowed for variation and customization of their product [10]. Established cigarette smokers who want to quit smoking may also try e-cigarettes as a cessation tool, in part because of the appealing flavors available [10].

The availability of e-cigarette flavors may promote youth experimentation [3, 7], as well as encourage relapse to a nicotine product among former smokers [8, 9]. Previous research has looked at reasons for using flavored products [7, 10], the health perceptions of flavored products [2], the effect of flavored products on future tobacco use among youth and young adults [8], and common flavors and number of flavors used among e-cigarette users [12–14], but, to our knowledge, no data are available on the popularity of specific flavor categories that are used in combination with each other. This analysis aims to assess the use of flavored e-cigarettes, in particular the use of multiple flavors, among nationally-representative samples of adults and youth in the US from Wave 2 of the Population Assessment of Tobacco and Health (PATH) Study.

## Methods

The PATH Study is a nationally representative, longitudinal cohort study of tobacco use and its health effects developed by the National Institutes of Health (NIH) and the FDA [15]. Its sample population aims to represent all noninstitutionalized US population, 12 years of age or older [15]. The PATH Study Wave 2 Youth (12–17 years old) and Adult (18+ years old) public-use data files were analyzed. Data were collected between October 23, 2014 and October 30, 2015 [15, 16]. Wave 2 included additional measures that allowed participants to identify use of a flavored tobacco product as well as specific flavor categories for each product used, including e-cigarettes. While the Wave 2 survey assessed use of e-cigarettes and other electronic nicotine delivery systems (ENDS), analyses were restricted to participants who reported use of an e-cigarette, not exclusive to use of other ENDS or other tobacco products. The prevalence of e-cigarette use in the past 30 days for the youth population was 3.1%, and 6.7% for the adult population [17].

### Study population

The study population for this paper included youth (12–17 years) and adults (18 years and older) who reported using any form of e-cigarettes on at least one day in the past 30 days. These participants were asked “In the past 30 days, were/was any of the e-cigarettes/e-cigarette cartridges/e-liquid you used flavored to taste like menthol, mint, clove, spice, fruit, chocolate, alcoholic drinks, candy or other sweets?” If participants reported yes, they were asked, “Which flavor was it? If multiple flavors were mixed together, choose all that apply.” The response options provided flavor categories including menthol/mint, clove/spice, fruit, chocolate, an alcoholic drink, candy/other sweets, or some other flavor. The final analysis included 415 youth participants and 2,123 adult participants.

### Statistical analysis

Prevalence of top flavor combinations, as well as flavors used alone, was assessed among youth and adults past 30-day e-cigarettes users. If participants responded that their e-cigarette was not flavored, it was assumed that their e-cigarette was tobacco flavor or not flavored at all. Analyses were conducted using Wave 2 study weights in Stata 14 software (StataCorp, 2011). Weighted percentages and 95% confidence intervals (95%CI) are reported. Multinomial logistic regressions were used to compare past 30-day youth and adult e-cigarette users. Models were adjusted for gender and race, and the adjusted odds ratios are presented.

## Results

### Youth

Among youth who reported using any form of e-cigarettes on at least one day in the past 30 days and provided flavor data, 80.8% were Non-Hispanic white, 57.0% were male, 80.4% were 15 to 17 years of age, and 61.4% were in high school (see **Table 1**). More than three-fourths of past 30-day youth users reported using a flavored e-cigarette (79.3%, 95CI: 74.5%, 83.3%). Of those who reported using a flavored e-cigarette, 40.6% (95CI: 35.7%, 45.6%) reported using a product that tasted like only one of the flavor categories (see **Table 1**). Fruit was the most popular individual flavor category chosen (55.0%; 95CI: 46.6%, 63.0%), followed by candy/other sweets (21.0%, 95CI: 14.5%, 29.3%); alcoholic drinks was the least popular individually-reported flavor category (1.1%, 95CI: 0.3%, 4.1%; see **Table 2**). There were 38.7% (95CI: 33.5%, 44.1%) past 30-day youth users who reported using some combination of these flavor categories (see **Table 1**). Fruit and candy/other sweets was the most common flavor combination (29.5%, 95CI: 22.4%, 37.7%), followed by fruit, candy/other sweets, and some other flavor (7.9%, 95CI: 4.0%, 15.3%). Though listed as an individual flavor choice, clove/spice was not reported in the top 10 flavor category combinations (see **Table 3**).

**Table 1:** Demographic characteristics of past 30 day adult and youth e-cigarette users who provided flavor data in the PATH Study Wave 2.

		Adult Past 30-day E-cigarette User	Youth Past 30-day E-cigarette User	p-value
		N = 2,123	N = 415	
**Gender**, N(%)				0.1318
	Male	1058 (52.4)	237 (57.0)	
	Female	1081 (47.6)	177 (43.0)	
**Race/Ethnicity**, N(%)				0.0348
	Non-Hispanic White	1649 (80.6)	314 (80.8)	
	Non-Hispanic Black	207 (9.7)	21 (6.2)	
	Other	240 (9.7)	64 (13.0)	
**E-cigarette Flavor Categories**, N(%)				<0.0001
	Tobacco/Unflavored	603 (31.0)	42 (10.9)	
	1 Flavor Category	921 (42.8)	171 (40.6)	
	2+ Flavor Categories	505 (21.8)	158 (38.7)	
	Don’t Know	94 (4.4)	44 (9.8)	

Abbreviations: LB = Lower 95% Confidence Bound, UB = Upper 95% Confidence Bound

**Table 2:** Prevalence of the most common to least common flavor categories among adult and youth respondents that reported using only one in the PATH Study Wave 2.

Adult Past 30-day E-cigarette User				Youth Past 30-day E-cigarette User			
N = 921				N = 171			
	%	LB	UB		%	LB	UB
**Menthol/Mint**	37.4	33.5	41.5	**Fruit**	55.0	46.6	63.0
**Fruit**	31.2	28.2	34.4	**Candy/Other Sweets**	21.0	14.5	29.3
**Candy/Other Sweets**	16.2	13.5	19.4	**Other Flavor**	12.8	7.7	20.5
**Other Flavor**	11.3	9.0	14.0	**Menthol/Mint**	6.1	3.0	11.7
**Chocolate**	2.2	1.2	3.9	**Clove/Spice**	2.1	0.7	6.1
**Alcoholic Drinks**	1.2	0.7	2.0	**Chocolate**	2.1	0.8	5.5
**Clove/Spice**	0.5	0.3	1.1	**Alcoholic Drinks**	1.1	0.3	4.1

Abbreviations: LB = Lower 95% Confidence Bound, UB = Upper 95% Confidence Bound

**Table 3:** Prevalence of the top 10 self-reported flavor category combinations reported among adults and youth who reported using 2 of more flavor categories in the PATH Study Wave 2.

Adult Past 30-day E-cigarette User				Youth Past 30-day E-cigarette User			
N = 505				N = 158			
	%	LB	UB		%	LB	UB
**Fruit and Candy/Other Sweets**	29.9	24.9	35.3	**Fruit and Candy/Other Sweets**	29.5	22.4	37.7
**Menthol/Mint, Fruit, and Candy/Other Sweets**	10.5	7.7	14.3	**Fruit, Candy/Other Sweets, and Other**	7.9	4.0	15.3
**Menthol/Mint and Fruit**	9.8	7.4	12.8	**Fruit and Other**	7.7	4.4	13.2
**Fruit, Candy/Other Sweets, and Other**	7.2	5.1	10.1	**Menthol/Mint, Fruit, and Candy/Other Sweets**	7.3	4.7	11.1
**Fruit and Other**	4.4	2.8	6.8	**Menthol/Mint and Fruit**	5.8	2.5	12.9
**Menthol/Mint and Candy/Other Sweets**	4.2	2.5	6.7	**Fruit, Chocolate, and Candy/Other Sweets**	4.7	2.3	9.4
**Menthol/Mint and Other**	3.2	1.8	5.9	**Menthol/Mint, Fruit, Candy/Other Sweets, and Other**	4.3	1.9	9.3
**Fruit, Chocolate, and Candy**	2.7	1.6	4.4	**Candy/Other Sweets and Other**	3.3	1.3	7.9
**Menthol/Mint, Fruit, Candy/Other Sweets, and Other**	2.1	1.0	4.1	**Menthol/Mint, Fruit, Alcoholic Drinks, and Candy/Other Sweets**	2.2	0.6	7.1
**Candy/Other Sweets and Other**	2.0	1.0	4.0	**Menthol/Mint, Fruit, Chocolate, Alcoholic Drinks, and Candy/Other Sweets**	1.9	0.6	5.7

Abbreviations: LB = Lower 95% Confidence Bound, UB = Upper 95% Confidence Bound

### Adults

Among adults who were past 30-day users of e-cigarettes and provided flavor data, 80.8% were Non-Hispanic white, 52.3% were male, 35.5% were 35 to 54 years of age, and 39.2% had some college or an Associate’s degree (see **Table 1**). Nearly two-thirds of past 30-day adult users of e-cigarettes (64.6%, 95CI: 62.0%, 67.2%) reported using a flavored product. Some reported using a product that tasted like only one flavor category (42.8%, 95CI: 40.4%, 45.3%), while fewer reported some combination of these flavor categories (21.8%, 95CI: 19.8%, 24.0%; see **Table 1**). Menthol/mint flavor was the most commonly reported individual flavor category among adult past 30-day users (37.4%, 95CI: 33.5%, 41.5%), with fruit being the second most common among this group (31.2%, 95CI: 28.2%, 34.4%; see **Table 2**). Clove/spice flavor was the least popular individually-reported flavor category (0.5%, 95CI: 0.3%, 1.1%; see **Table 2**) among past 30-day adult users of e-cigarettes. Among adults reporting more than one flavor category, the most common combination was fruit and candy/other sweets (29.9%, 95CI: 24.9%, 35.3%) followed by menthol/mint, fruit and candy/other sweets (10.5%, 95CI: 7.7%, 14.3%). Similar to the findings for youth, when examining combination flavor categories, clove/spice flavor category was not reported in the top 10 self-reported flavor category combinations (see **Table 3**).

### Comparing past 30 day youth and adult e-cigarette users

Past 30-day youth and adult users of e-cigarettes differed significantly on type of flavor/number of flavor categories used (p ≤ 0.001; see **Table 1**). Compared to adults, past 30-day youth e-cigarette users were significantly more likely to report using a flavored e-cigarette(s) than a tobacco or unflavored e-cigarette (1 Flavor: 2.83, 95%CI: 1.99, 4.03; 2+ Flavors: 5.26, 95%CI: 3.60, 7.68; see **Table 4**). Furthermore, youth were significantly less likely to report using a single flavor category than two or more flavor categories (0.53, 95%CI: 0.41, 0.70) compared to adults. When comparing flavor categories reportedly used in the past 30 days between adults and youth, youth were significantly more likely than adults to report using an e-cigarette flavored like fruit (2.11, 95%CI: 1.55, 2.87) than any other flavor, and significantly less likely to report using and e-cigarette flavored like menthol/mint (0.14, 95%CI: 0.06, 0.32) than any other flavor. Although not statistically significant, youth were more likely to report using a candy/other sweet flavored e-cigarette than any other flavor compared to adults (1.25, 95%CI: 0.79, 1.99). There was no difference between youth and adults who selected some other flavor in relation to the other flavor category options. Finally, the most common flavor category combination for both past 30-day youth and past 30-day adult e-cigarette users was a fruit flavor with a candy/other sweets flavor, with youth significantly more likely to report them together (1.90, 95CI: 1.26, 2.88) than any other flavor category combination.

**Table 4:** Comparison of flavor category use among past 30 day adult and youth e-cigarette users in the PATH Study Wave 2.

		1 Flavor Category v. Tobacco/Unflavored		2+ Flavor Categories v. Tobacco/Unflavored		Don't Know v. Tobacco/Unflavored	
		N = 1,092		N = 663		N = 138	
		Crude	Adjusted^a^	Crude	Adjusted^a^	Crude	Adjusted^a^
**Age Group**							
	Adult	Ref	Ref	Ref	Ref	Ref	Ref
	Youth	**2.69 (1.92, 3.78)**	**2.83 (1.99, 4.03)**	**5.05 (3.50, 7.29)**	**5.26 (3.60, 7.68)**	**6.37 (4.04, 10.05)**	**6.21 (3.84, 10.03)**
**Gender**							
	Male	Ref	Ref	Ref	Ref	Ref	Ref
	Female	1.19 (0.96, 1.47)	**1.33 (1.02, 1.73)**	0.98 (0.80, 1.20)	1.17 (0.94, 1.46)	0.65 (0.40, 1.04)	0.67 (0.40, 1.13)
**Race/Ethnicity**							
	Non-Hispanic White	Ref	Ref	Ref	Ref	Ref	Ref
	Non-Hispanic Black	**2.11 (1.41, 3.18)**	**2.39 (1.52, 3.76)**	1.38 (0.84, 2.29)	1.76 (1.00, 3.10)	**2.29 (1.16, 4.53)**	**2.29 (1.12, 4.65)**
	Other	1.40 (0.89, 2.20)	1.40 (0.85, 2.32)	1.31 (0.87, 1.97)	1.58 (0.97, 2.58)	**2.46 (1.18, 5.16)**	**2.21 (1.02, 4.79)**

NOTE: Weighted odds ratios (95% Confidence intervals) are presented. The referent category of the dependent variable, Tobacco/Unflavored, had 654 observations. Bolded point estimates indicate statistical significance at p<0.05.

a: Adjusted for gender and race/ethnicity

## Discussion

This study identified the most popular e-cigarette flavor categories that are used alone, as well as in combination with other flavor categories, among past 30-day youth and adult e-cigarette users. Most e-cigarettes users, youth and adult, reported using an e-cigarette that was flavored to taste like menthol, mint, clove, spice, fruit, chocolate, alcoholic drinks, candy or other sweets, or another flavor. About 41% of youth reported using one flavor category, and nearly 40% using more than one flavor category in combination. On the other hand, about 43% of adults reported using one flavor category but only about 22% reported using two or more flavor categories. Among youth and adults, fruit and candy/other sweets flavors were found in the top three individually-reported flavor categories. These findings are consistent with previous reports on e-cigarette flavor preference [12–14]. Of the top ten flavor category combinations reported among past 30-day youth and adult users, most of the flavor combinations are common in both study populations with fruit and candy/other sweets being the most popular flavor categories chosen together. However, comparison to prior studies (e.g., 14) is hampered by differences in type and number of flavor categories presented to participants, including separation of options for menthol and mint, and presentation of tobacco as a discrete flavor option (rather than the assumed default). These differences would complicate a direct comparison between studies of total number of flavors reported by participants. This argues for greater consistency in presentation of flavor choices in surveys.

It may be difficult to separate nominal ‘flavor’ from properties such as sweetness [12, 18–22]. Taste and smell can serve as conditioned reinforcers and, via incentive salience mechanisms, may increase motivation to consume nicotine. For example, in young adult smokers (age 18–30), fruit and dessert-flavored ENDS have been found to be more satisfying and showed greater reinforcing value in a laboratory task [21]. Tobacco-flavored ENDS were perceived as least sweet and least liked, with flavor liking ratings increasing directly with sweetness in other laboratory work [19]. In the current study, fruit flavor (typically sweeter) was seen in nine out of the top ten flavor combinations among both youth and adults, while clove/spice flavor (typically more bitter) was not seen in any of the top ten flavor category combinations, consistent with other population and laboratory research [19, 21, 23]. Of note, adults were more likely to have menthol or mint as a primary flavor, or as one of multiple flavors endorsed, which may represent a carryover from associations with tobacco cigarettes. This suggests that future research should focus on the composition of the ENDS liquid/vapor to disentangle the contributions of sweeteners and flavorants.

This analysis has some limitations to note. First, flavor data were self-reported based on a check-all list, so we cannot distinguish between using mixed flavored e-liquids from using two distinct flavored e-liquids at different times, nor using two different flavors under the same category. Second, we do not have data on source of liquid and the exact composition of a flavored liquid may vary from brand to brand. Finally, due to the select sample that was asked the e-cigarette flavor question in the Wave 2 PATH survey (i.e., participants who reported not smoking a flavored e-cigarette were skipped out of the question describing flavor categories), we cannot determine the proportion of participants who may have used tobacco flavor or an unflavored e-liquid in combination with a characterizing flavor e-liquid. We estimate that this potential limitation would likely result in an underestimation of flavor use, thereby biasing our results towards the null. At the same time, PATH is nationally representative and a longitudinal cohort study of tobacco use among both youth and adults in the US, and thus is most likely the best source for data on flavor preferences in the population. These limitations point to the need for flavor questionnaire refinement, as suggested by others (18). Allowing for participants to write in names of flavors, and having a rule-based classification system, could improve accuracy [24]. However, some names may not reflect the underlying flavors used to create the e-liquid, necessitating further investigation. As manufacturers are now required to register and provide product listings under the 2016 FDA Deeming rule, an accessible database of product names and primary flavors is feasible and could be helpful.

E-cigarettes are appealing to both youth and adults because of the variety of flavors that allow for customization. Many e-cigarette users are not only using one flavor but combinations of flavors, which imply potential exposure to a number of chemical compounds. Prudent regulation, short of extending the ban on characterizing cigarette flavors to other products, could involve restricting the use of flavors that are potentially harmful to health. Flavors such as fruit and cinnamon have been shown to contain respiratory toxicants [3]. Although some have reported using e-cigarettes as a tool to quit smoking [11], the wide variety of flavors available, specifically sweet flavors, and the ability to “mix-and-match” flavors, may sustain e-cigarette use among youth and adults [7, 10]. Understanding patterns of flavor preference provides important insights into potential toxicant exposure pathways and also may implicate pathways involved in the development or maintenance of nicotine dependence.
